# Effects of high-intensity interval inspiratory muscle training on diaphragm function and quality of life among tracheostomized patients: study protocol for a randomized controlled trial

**DOI:** 10.1186/s13063-025-09334-3

**Published:** 2025-12-03

**Authors:** Fangting Chen, Mingxue Xia, Lijuan Li, Yangyang Lin, Yafei Wang, Huiting Feng, Weiming Wang, Jianlin Ou, Yuling Wang

**Affiliations:** 1 Department of Rehabilitation Medicine, The Sixth Affiliated Hospital, Sun Yat-Sen University, Guangdong, China; 2 Biomedical Innovation Center, The Sixth Affiliated Hospital, Sun Yat-Sen University, Guangdong, China; 3 Guangdong Provincial Clinical Research Center for Rehabilitation Medicine, Guangzhou, China; 4 Department of Rehabilitation Medicine, The First Affiliated Hospital of Jinan University, Guangdong, China

**Keywords:** High-intensity interval inspiratory muscle training, Tracheostomy, Diaphragm function, Decannulation, Quality of life

## Abstract

**Background:**

Patients who undergo tracheostomy often experience respiratory muscle dysfunction and reduced airway clearance capacity, leading to reduced quality of life. While inspiratory muscle training (IMT) is effective at improving respiratory outcomes among mechanically ventilated patients, evidence about its clinical utility and safety, especially in tracheostomized populations, is limited. Moreover, the optimal IMT protocol for this patient cohort remains undefined. The aim of this study is to evaluate the effects of high-intensity interval inspiratory muscle training (HI-IMT) on diaphragmatic function, airway clearance capacity, anxiety levels, decannulation rates, quality of life, and safety outcomes among tracheostomy patients.

**Methods:**

This is a single-center, single-blind, randomized controlled trial. Seventy tracheostomized patients (aged 40–70 years, with tracheostomy for 1–3 months) will be randomly assigned to either the HI-IMT group or the control group. The HI-IMT group will receive IMT at 50% of the maximal inspiratory pressure (MIP), 30 breaths/day, 5 days/week for 2 weeks. The control group will receive sham training, which will involve performing the same breathing cycles without any applied resistance. Both groups will receive standard care, including pharmacological therapy, rehabilitative nursing, and basic rehabilitation exercises. Diaphragmatic function, airway clearance ability, anxiety levels, and quality of life will be assessed using the MIP% predicted (primary outcome) and peak inspiratory flow (PIF), the Semi-Quantitative Cough Strength Score (SCSS), the standardized Zung Self-Rating Anxiety Scale (SAS), and the Tracheostomy-Specific Quality of Life (TQOL) scale. Additionally, decannulation rates and adverse events will be systematically recorded throughout the study period. Data will be analyzed using parametric or non-parametric tests for unadjusted between-group comparisons. All analyses will adhere to the intention-to-treat principle, with a *p*-value < 0.05 set for statistical significance.

**Discussion:**

This study is expected to provide valuable evidence about the efficacy and safety of HI-IMT in improving diaphragmatic function and overall outcomes among tracheostomized patients. These findings may contribute to the development of standardized respiratory rehabilitation protocols tailored to this patient population.

**Trial registration:**

Chinese Clinical Trial, ChiCTR2500099091. Registered on 18 March 2025. https://www.chictr.org.cn/bin/project/edit?pid=265948.

**Supplementary Information:**

The online version contains supplementary material available at 10.1186/s13063-025-09334-3.

## Administrative information

Note: the numbers in curly brackets in this protocol refer to SPIRIT checklist item numbers. The order of the items has been modified to group similar items (see http://www.equator-network.org/reporting-guidelines/spirit-2013-statement-defining-standard-protocol-items-for-clinical-trials/).

**Table:** 

Title {1}	Effects of high-intensity interval inspiratory muscle training on diaphragm function and quality of life among tracheostomized patients: Study protocol for a randomized controlled trial
Trial registration {2a and 2b}	The trial was registered in the Chinese Clinical Trial Registry with the code ChiCTR2500099091 and was approved on 18 March 2025. https://www.chictr.org.cn/bin/project/edit?pid=265948.
Protocol version {3}	Version 2 of 09-7-2024
Funding {4}	This study is funded by Guangdong Provincial Clinical Research Center for Rehabilitation Medicine (2023B110003).
Author details {5a}	Fangting Chen, Mingxue Xia, Lijuan Li, Yangyang Lin, Yafei Wang, Huiting Feng, Weiming Wang, Yuling Wang: Department of Rehabilitation Medicine, The Sixth Affiliated Hospital, Sun Yat-sen University; Biomedical Innovation Center, The Sixth Affiliated Hospital, Sun Yat-sen University; Guangdong Provincial Clinical Research Center for Rehabilitation Medicine. Jianlin OU: Department of Rehabilitation Medicine, The First Affiliated Hospital of Jinan University.
Name and contact information for the trial sponsor {5b}	Yuling Wang, wangyul@mail.sysu.edu.cn
Role of sponsor {5c}	This is an investigator initiated clinical trial. Therefore, the funders had no involvement in the study design, data collection, analysis, interpretation, or preparation of the manuscript.

## Introduction

### Background and rationale {6a}

Tracheostomy is a critical intervention for maintaining airway patency in critically ill patients [1]. Epidemiological data indicate that approximately 13% to 35% of these individuals undergo tracheostomy during intensive care treatment [2–4]. However, this procedure disrupts normal respiratory physiology, leading to diaphragmatic weakness, diminished cough effectiveness, and increased risk of pulmonary infections [5–7]. These complications are associated with delayed decannulation, prolonged hospitalization, and reduced overall quality of life [8].

In current clinical practice, respiratory rehabilitation for tracheostomized patients relies primarily on traditional methods, such as abdominal (diaphragmatic) breathing, pursed-lip breathing, candle-blowing exercises, paper strip resistance training, manual hyperinflation technique, or use of incentive spirometers [9–11]. However, these approaches often do not allow accurate assessment of respiratory muscle strength and fail to provide personalized training protocols. Since respiratory muscles are part of the skeletal muscle system, exercises to strengthen them should adhere to the principle of overload [12]. Nonresistive training modalities may not be sufficient to fully enhance patient lung function [13–15].

Inspiratory muscle training (IMT), which specifically targets the diaphragm and accessory inspiratory muscles, has demonstrated efficacy in mechanically ventilated patients [16]. A systematic review including 28 studies (*n* = 1184) confirmed that IMT increases inspiratory muscle strength and facilitates earlier weaning from mechanical ventilation in intensive care settings [17]. Nevertheless, most available evidence pertains to intubated patients, and there are limited data specific to tracheostomized populations [18–22]. Importantly, tracheostomy alters respiratory mechanics, such as by reducing airway resistance and impairing the generation of negative inspiratory pressure, requiring tailored training approaches. Furthermore, the use of conventional threshold-loading devices in tracheostomized patients has disadvantages, including ceiling effects and mismatches between pressure and volume during high-resistance training [23, 24].

The POWERbreathe KH2 is a novel inspiratory muscle training device that uses tapered flow resistive loading (TFRL), potentially overcoming the limitations of traditional threshold loading (TL) methods [25]. Unlike TL, TFRL introduces an initial threshold followed by a velocity-dependent decline in resistance, resembling isokinetic training of the musculature of the limb [23, 26, 27]. This configuration minimizes pressure-volume conflicts and enables real-time monitoring of inspiratory pressure and flow, thereby allowing precise dosage adjustments. Preliminary case studies and small-sample investigations suggest that the device is safe and feasible for use with tracheostomized patients, although robust evidence from randomized controlled trials is currently lacking [28, 29].

Moreover, the optimal IMT protocol for tracheostomized individuals remains to be established. As tracheostomized patients often have weak inspiratory muscles, they are unlikely to tolerate sustained loading of the respiratory muscles without distressing fatigue. High-intensity interval inspiratory muscle training (HI-IMT), which is characterized by high-intensity and low-repetition protocols, represents a promising therapeutic strategy for this population. This modality can optimize therapeutic efficacy while minimizing treatment-induced discomfort. This approach has been used successfully in numerous trials [30–33].

### Objectives {7}

The primary objective of this study is to evaluate the efficacy of HI-IMT in improving the diaphragmatic function of patients undergoing tracheostomy. The secondary objectives include investigating the effects of HI-IMT on airway clearance capacity, anxiety levels, quality of life, decannulation rates, and safety outcomes.

### Trial design {8}

**Fig. 1:**
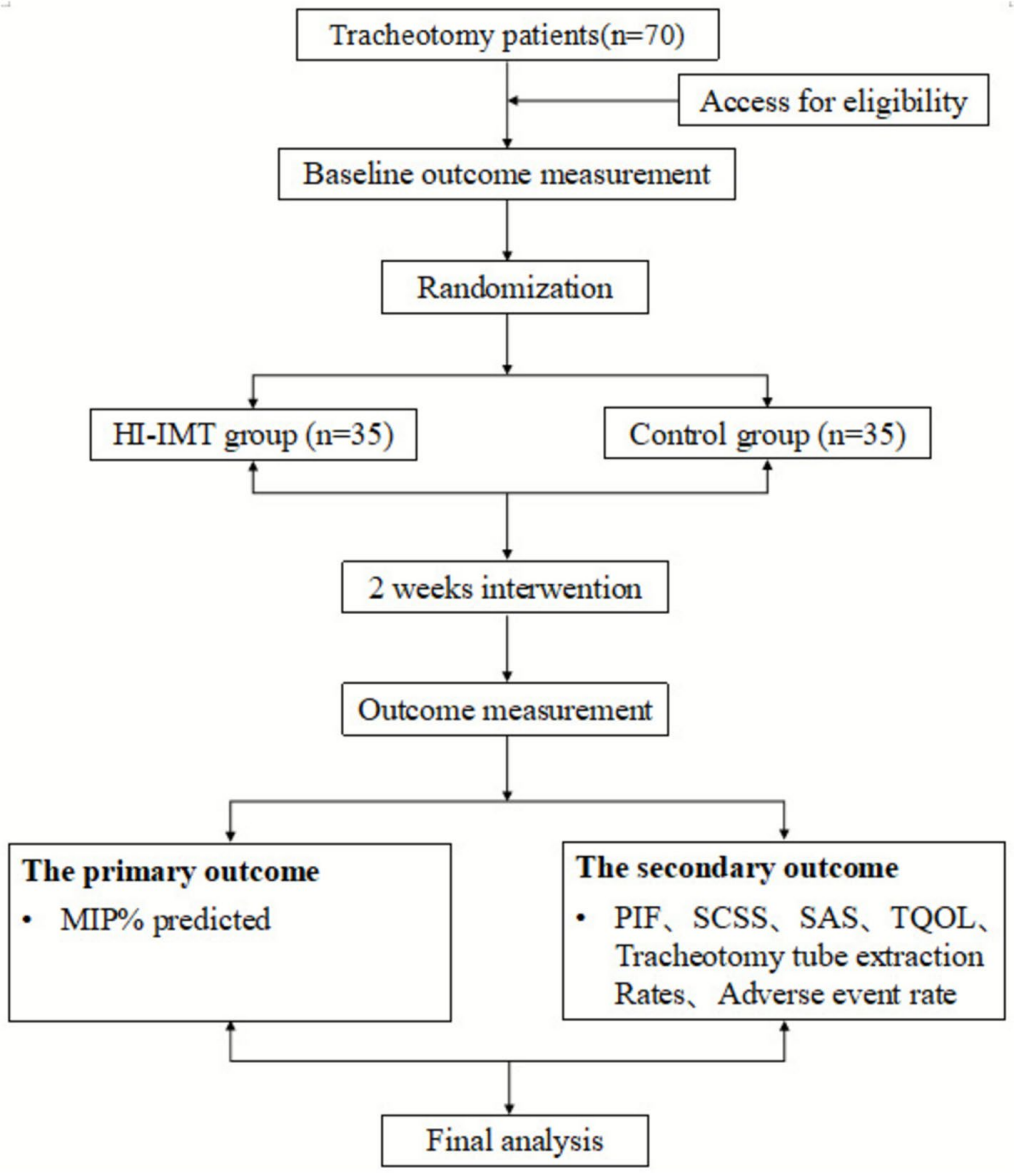
Flow chart of the study

This is a single-center, randomized, single-blind, parallel-group, exploratory clinical trial. A total of 70 tracheostomized patients will be enrolled and randomly allocated at a 1:1 ratio to either the HI-IMT intervention group or the control group. The study procedure is shown in Fig. 1.

## Methods: participants, interventions, and outcomes

### Study setting {9}

This study will be conducted at the Sixth Affiliated Hospital of Sun Yat-sen University. All the evaluations and training sessions will take place either in the rehabilitation medicine treatment room or at the patient’s bedside, depending on individual clinical needs.

### Eligibility criteria {10}

Inclusion criteria:

1. Patients aged 40–70 years;
2. Patients undergoing tracheostomy for 1–3 months, with a daily suction frequency of ≥ 10 times and MIP% predicted ≤ 30%;
3. Patients with a 7.5 mm diameter tracheostomy tube;
4. Patient with stable vital signs and who are conscious, cognitively intact, and able to understand and cooperate with training, achieve a score of 5 on the Standardized Five Questions (S5Q) [34];
5. Patients without evidence of airway collapse or stenosis confirmed by fiberoptic bronchoscopy;
6. Patients with oxygen saturation (SpO₂) ≥ 93% on room air, respiratory rate ≤ 25 breaths/min, blood pressure between 90 and 180 mmHg, heart rate between 60 and 120 beats/min, and body temperature between 36.5 and 38.5 °C;
7. Patients who are willing to participate in the study and provide signed informed consent.

Exclusion criteria:

1. Patients with a metal tracheostomy tube, other non-cuffed tracheostomy tubes, or fenestrated tubes;
2. Patients with a diagnosis of progressive neuromuscular disease;
3. Patients with a contraindications to IMT, including rib fractures, pulmonary bullae, or acute asthma exacerbation;
4. Patients with unhealed tympanic membrane perforation, other significant ear disorders, or evidence of aspiration;
5. Patients with severe comorbidities affecting major organ systems, such as severe cardiac dysfunction (NYHA Class III/IV heart failure), severe hepatic insufficiency (Child-Pugh class B/C), or end-stage renal disease requiring dialysis;
6. Patients for whom suctioning is required more frequently than once per hour.

### Who will obtain informed consent? {26a}

A designated member of the research team will provide each eligible participant with a comprehensive explanation of the study’s objectives, procedures, potential risks and benefits, and any expected discomfort. The participants will be clearly informed that their involvement is entirely voluntary and that they may withdraw from the study at any time without any impact on their future medical care or treatment. Written informed consent will be obtained using an ethics committee–approved consent form prior to the initiation of any study-related procedures.

### Additional consent provisions for the collection and use of participant data and biological specimens {26b}

Not applicable, as no biological samples will be collected or stored for this study.

## Interventions

### Explanation for the choice of comparators {6b}

The control group will undergo placebo inspiratory muscle training (IMT) using the same POWERbreathe KH2 device as the HI-IMT group; however, no resistance load will be applied. The participants in both groups will receive standard medical care, as detailed in item 11a.

### Intervention description {11a}

#### Conventional medical interventions

Conventional medical interventions include pharmacological treatment, rehabilitation nursing, physical therapy, and dysphagia management. Pharmacotherapy includes the administration of anti-infectives, mucolytics, and nutritional support, as well as the regulation of electrolyte balance [35]. Rehabilitation nursing involves airway humidification, tracheal suctioning, oral hygiene, and monitoring of tracheal cuff pressure. Physical therapy integrates postural drainage, percussion techniques, and the active cycle of breathing technique (ACBT), which are typically delivered in 20-min sessions. Dysphagia management includes neuromuscular electrical stimulation (NMES) to enhance pharyngeal constriction, along with orofacial sensory–motor training to improve swallowing coordination, which are also administered in 20-min sessions.

#### HI-IMT group

**Fig. 2:**
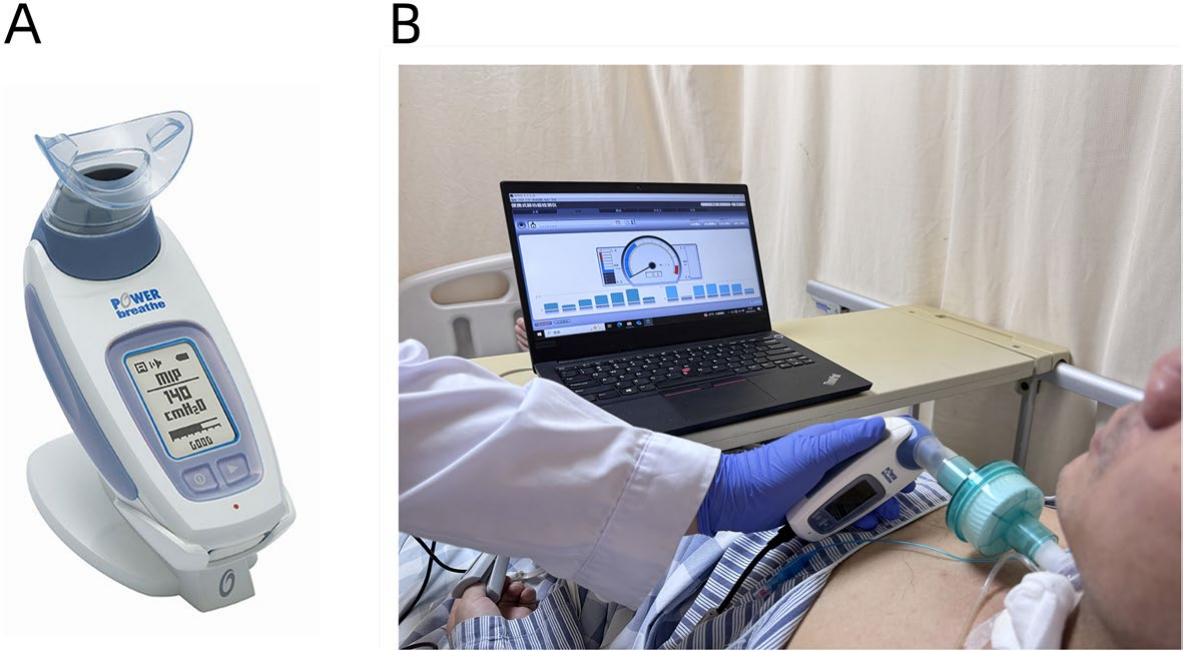
High-intensity interval inspiratory muscle training. **A** POWERbreathe KH2 device; **B** subjects undergoing high-intensity interval inspiratory muscle training in bed

In addition to conventional care, participants in the HI-IMT group will receive inspiratory muscle training under the guidance of a trained physiotherapist using the POWERbreathe KH2 device (Fig. 2). Prior to the intervention, the MIP will be measured with the tracheal cuff inflated to ensure accurate loading. A wet heat exchanger will be connected between the tracheostomy site and the POWERbreathe KH2. Patients will be instructed to exhale slowly for at least 6 s until the residual volume is reached, followed by maximal inhalation lasting at least 1.5 s. Each patient will perform at least three MIP trials, with 2-min rest intervals between attempts. The highest value will be recorded as the MIP.

The initial training load will be set at 50% of the MIP and will be increased by 2 cmH₂O daily. The MIP will be re-measured at the beginning of the second week, and the new training load will be determined by comparing 50% of the newly obtained MIP value with the training load achieved at the end of the first week. The starting load for the second week will be set to the higher of these two values. Each session will consist of 30 breaths divided into 5 sets of 6 breaths each, with 2-min rest intervals between sets. The participants will follow a pattern of rapid inhalation and slow exhalation. Sessions will last approximately 20 min and will be conducted once daily, 5 days per week, over a 2-week period.

Training will be paused if signs of respiratory insufficiency or hemodynamic instability are observed, defined as any of the following: respiratory rate ≥ 35 breaths/min (or > 50% above baseline), SpO₂ ≤ 90%, systolic blood pressure ≥ 180 mmHg or ≤ 80 mmHg, heart rate ≥ 140 bpm or > 20% above baseline, paradoxical breathing, agitation, depression, hemoptysis, arrhythmias, or diaphoresis [36]. If any of these criteria are met, the training intensity in the next session will not increase and will remain at the previously tolerated level.

#### Control group

The control group will follow the same evaluation protocol and follow the same training program but without any applied resistance.

### Criteria for discontinuing or modifying allocated interventions {11b}

During each training session, therapists will closely monitor vital signs and subjective symptoms. The discontinuation criteria are outlined in item 11a. Any adverse events will be documented in the daily training log. If a participant meets two or more discontinuation criteria on three consecutive training days, they will be withdrawn from the study.

### Strategies to improve adherence to interventions {11c}

The POWERbreathe KH2 device is equipped with data logging functionality to record each session’s performance, ensuring objective tracking of adherence. Additionally, both IMT and placebo interventions will be provided to participants at no cost as a motivational incentive.

### Relevant concomitant care permitted or prohibited during the trial {11d}

No specific concomitant treatments are prohibited during the trial, except for concurrent participation in other clinical studies that could interfere with IMT outcomes.

### Provisions for posttrial care {30}

No trial-related harm is anticipated. As such, no compensation or special posttrial care arrangements are applicable.

### Outcomes {12}

Primary and secondary outcomes will be assessed by trained research staff who remain blinded to treatment group allocation throughout the study.

#### Primary outcome

The primary outcome of this study is the change in the MIP % predicted from baseline to the post-intervention timepoint (Week 2). MIP is a widely recognized and reliable indicator for assessing diaphragmatic function [37, 38]. Raw MIP scores were standardized according to the method of Evans and Whitelaw (males = 120 **−** (0.41** × **age); females = 108 **−** (0.61** × **age)) [39]. To account for established variations in MIP attributable to age and gender, the results are expressed as a percentage of predicted values.

#### Secondary outcomes

The secondary outcomes include changes in peak inspiratory flow (PIF), airway clearance ability, anxiety levels, quality of life, the decannulation rate, and the incidence of adverse events.

##### PIF

The PIF serves as a key parameter reflecting the velocity of diaphragmatic contraction. Higher PIF values indicate an increased capacity to overcome airway resistance [40].

##### Airway clearance ability

The Semi-Quantitative Cough Strength Score (SCSS) [41] is a 6-point ordinal scale (0–5) that has been validated for the assessment of cough efficacy as follows: 0: no cough; 1: visualized cough (oral airflow present) without sound; 2: barely audible cough; 3: distinctly audible cough; 4: forceful cough; and 5: sustained vigorous coughing. The SCSS will be assessed during brief manual occlusion of the tracheostomy stoma.

##### Anxiety levels

Anxiety will be measured using the standardized Zung Self-Rating Anxiety Scale (SAS), which is a widely applied instrument with established psychometric properties [42].

##### Quality of life

Quality of life will be evaluated using the Chinese version of the Tracheotomy-Specific Quality of Life (TQOL) scale [43]. This instrument, which was adapted through rigorous cross-cultural validation, comprises four domains—physical functioning, somatic status, social/family interaction, and overall satisfaction—and has been demonstrated to have high reliability and content validity.

##### Tracheotomy tube extraction rates

**Fig. 3:**
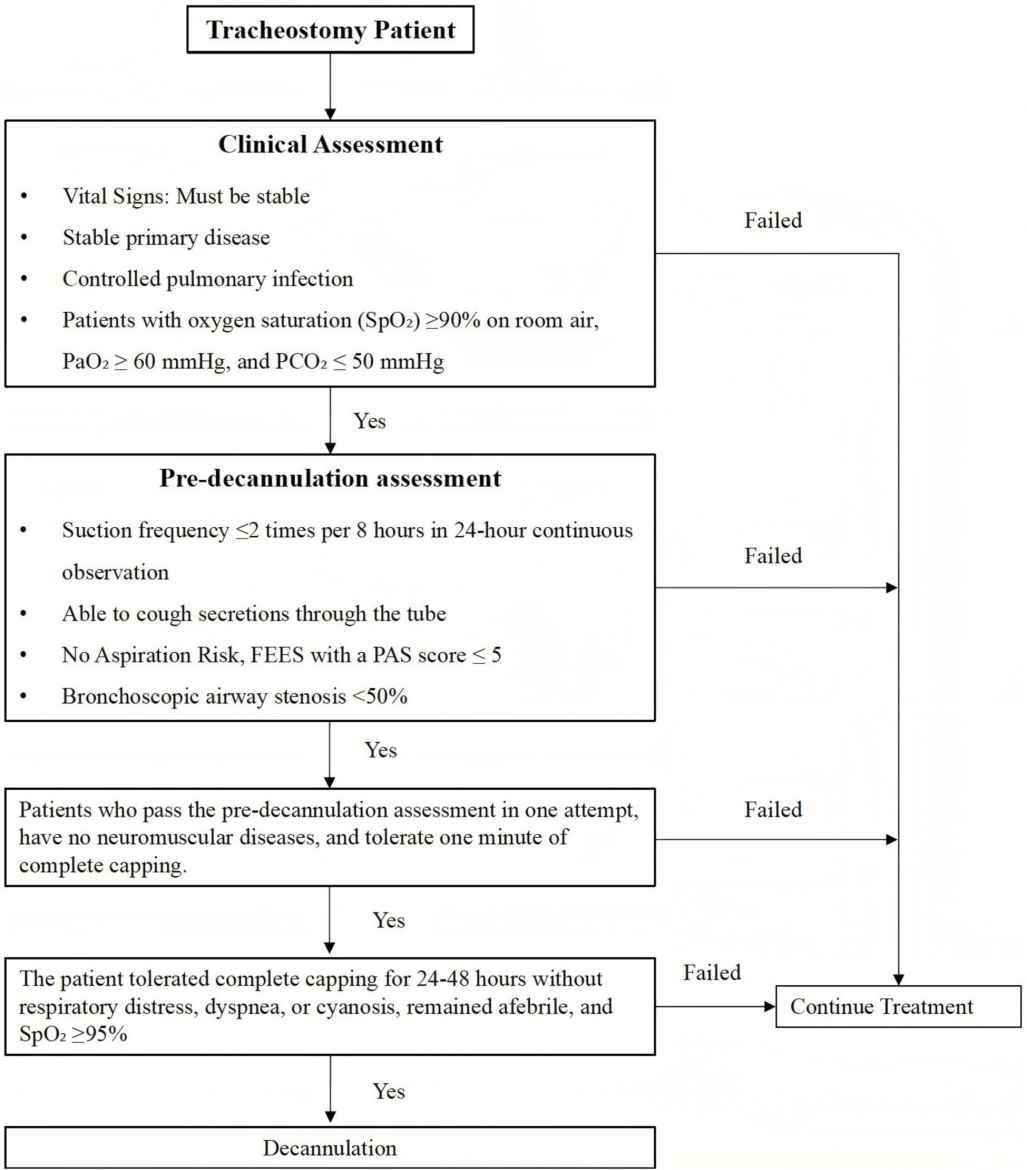
Patient management pathway for tracheostomy decannulation

The decannulation rate will be recorded for both the intervention and control groups over the 2-week intervention period. Decannulation decisions will be made by clinicians who will be blinded to patient allocation, following the evidence-based protocol of the Chinese Expert Consensus on Tracheotomy Decannulation in Adults (2023) [44]. The specific protocol is presented in Fig. 3.

### Participant timeline {13}

The details of the timeline are presented in Table 1.

**Table 1:**
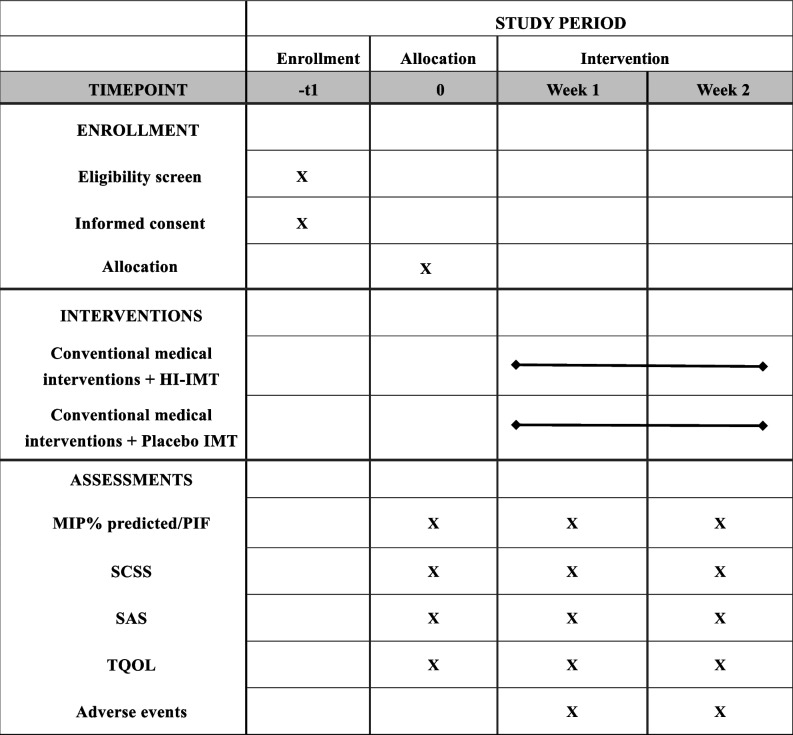
Schedule of enrollment, intervention, and assessments

### Sample size calculation {14}

The sample size was determined based on the primary outcome (change in MIP% predicted). Using data from Bissett et al.’s RCT where high-intensity IMT demonstrated a mean improvement of 17% (SD = 4%) versus 6% (SD = 3%) in controls [24], we conservatively estimated a mean difference of 8% between groups. To account for greater clinical heterogeneity in tracheostomized patients, we adopted a pooled SD of 9% (versus 3.54% in Bissett’s study). Using G*Power 3.1 with a two-tailed *t*-test (*α* = 0.05, power = 0.90, allocation ratio 1:1), the initial calculation indicated 28 participants per group (total *N* = 56). Considering a 20% attrition rate (based on Bissett’s reported 17% loss to follow-up), the adjusted sample size was 35 per group (total *N* = 70).

### Recruitment {15}

A total of 70 tracheostomized patients will be recruited from the Sixth Affiliated Hospital of Sun Yat-sen University. A multidisciplinary collaborative network will be established to facilitate screening and referral of eligible candidates on the basis of the predefined inclusion criteria. Recruitment announcements will be posted on the hospital’s public website. Neutral informational materials describing IMT as an experimental respiratory training technique will be available in inpatient wards and outpatient clinics. A dedicated study coordinator will conduct individual consultations with eligible candidates to explain the study objectives, procedures, and safety considerations; address any concerns; and assist with the informed consent process.

## Assignment of interventions: allocation

### Sequence generation {16a}

The randomization sequence will be generated using SPSS version 26.0 by a researcher who is not involved in trial operations or outcome assessments. A simple randomization strategy will be employed to assign participants in a 1:1 ratio to either the HI-IMT group or the control group.

### Concealment mechanism {16b}

Allocation concealment will be ensured through the use of sealed, opaque envelopes. Each envelope will contain the group assignment and will be opened by the study investigator immediately before the initiation of the intervention.

### Implementation {16c}

The randomization sequence will be prepared by an independent statistician who will not be involved in participant recruitment or intervention delivery. Clinical staff will enroll eligible participants, and an independent researcher will assign interventions on the basis of the sealed envelope system.

## Assignment of interventions: blinding

### Who will be blinded {17a}

This is a single-blind trial. The participants will be blinded to group assignment. Owing to the nature of the intervention, it is not feasible to blind the therapists conducting the training. However, outcome assessors and the data analyst will remain blinded to the group allocation to minimize bias during outcome analysis.

### Procedure for unblinding if needed {17b}

Unblinding will be permitted only if a serious adverse event occurs that requires immediate clinical intervention or after the completion of final statistical analyses. Any unblinding must be approved by the principal investigator and documented in the case report form (CRF).

## Data collection and management

### Plans for assessment and collection of outcomes {18a}

Two trained research personnel will be present during each outcome assessment who will be blinded to the participants’ group allocation: one responsible for guiding the participant and the other for real-time data documentation. Both assessors must have prior experience and standardized training in outcome evaluation. Data will be recorded directly on the CRF and signed and dated by both staff members. Demographic data will be collected during the initial session. Details of the outcome measures are provided in the “Outcomes {12}” section.

### Plans to promote participant retention and complete follow-up {18b}

During the first session, the participants will be clearly informed about the importance of attending all the scheduled sessions through the end of the study. Only those who are able to fully comply with the protocol will be enrolled. The importance of adherence will be emphasized throughout the intervention period. Participants who discontinue the study will be recorded as dropouts.

### Data management {19}

All the participant data will be documented on individual CRFs and deidentified using unique study codes. Personal identifiers will not be recorded. All the data will be securely stored in locked file cabinets that are accessible only to authorized members of the research team. No data will be shared.

### Confidentiality {27}

Data confidentiality procedures are outlined in the “Plans for assessment and collection of outcomes {18a}” and “Data management {19}” sections.

### Plans for collection, laboratory evaluation, and storage of biological samples for genetic or molecular analysis in this trial/future use {33}

Not applicable, as this study will not involve the collection, storage, or analysis of biological samples.

## Statistical methods

### Statistical methods for primary and secondary outcomes {20a}

Statistical analyses will be conducted in accordance with the CONSORT guidelines to ensure methodological transparency and rigor [45]. The primary analytical approach will follow the intention-to-treat (ITT) principle, including all randomized participants irrespective of adherence to the intervention. In cases where the percentage of missing data exceeds 20%, multiple imputation will be applied using predictive mean matching for continuous variables. Sensitivity analyses will also be conducted, comparing ITT and per-protocol (PP) populations. The PP population will consist of participants who complete at least 80% of the scheduled training sessions, enabling evaluation of result robustness.

The data will be analyzed with SPSS version 26.0. The normality of continuous variables will be assessed using the Shapiro–Wilk test and visual inspection of histograms. Between-group differences in the MIP will be analyzed with independent samples *t* tests (for normally distributed data) or Mann–Whitney *U* tests (for nonnormally distributed data). The secondary outcomes will be analyzed as follows: continuous variables will be compared between groups with t tests or Mann–Whitney *U* tests on the basis of distributional assumptions. Ordinal outcomes will be evaluated with Wilcoxon rank-sum tests. Binary outcomes will be analyzed with chi-square tests or Fisher’s exact tests for small sample sizes. Adverse events will be summarized descriptively by frequency and severity.

### Interim analyses {21b}

No interim analyses are planned owing to the short trial duration (2 weeks) and the exploratory nature of the study.

### Methods for additional analyses (e.g., subgroup analyses) {20b}

No subgroup analyses are planned.

### Methods in analysis to handle protocol non‑adherence and any statistical methods to handle missing data {20c}

An ITT approach will be used for all primary and secondary outcome analyses. Multiple imputation will be employed to address missing data, ensuring statistical validity.

### Plans to provide access to the full protocol, participant-level data and statistical code {31c}

The results will be published in journals.

## Oversight and monitoring

### Composition of the coordinating center and trial steering committee {5d}

The trial steering committee (TSC), which is composed of experienced professionals, is responsible for ensuring scientific rigor, ethical compliance, and operational integrity throughout the clinical trial. Its structure and functions are as follows:

1. Composition:
  A senior physician chairperson.
  Multidisciplinary medical experts (e.g., biostatistics, clinical practice).
  An independent ethics specialist.
2. Roles and responsibilities:
  Evaluate trial design, protocols, and informed consent documents for scientific validity and regulatory adherence.
  Monitor trial progress, safety outcomes, and ethical standards.
  Provide expert recommendations to resolve challenges and enhance trial quality.
  Safeguard participant rights and welfare through ongoing oversight.
  This streamlined framework maintains accountability while emphasizing collaborative oversight across scientific, ethical, and operational domains.

### Data monitoring committee (DMC) {21a}

No independent DMC is established, as this is a low-risk, short-term trial. Safety monitoring is performed by the coordinating center.

### Adverse event reporting and harms {22}

Although this study is associated with minimal known risks, participants are instructed to immediately notify the responsible physiotherapist of any adverse or serious events. The possible side effects may include fatigue, dizziness, or transient decreases in blood oxygen saturation. Any adverse events will be promptly reported to the Institutional Research Ethics Committee by the designated principal investigator.

### Frequency and plans for auditing trial conduct {23}

The Trial Steering Committee will conduct biweekly audits throughout the study period. During these meetings, the committee will review participant recruitment progress, the informed consent process, adherence to the study protocol, and all recorded adverse events.

### Plans for communicating important protocol amendments to relevant parties (e.g., trial participants, ethical committees) {25}

Any modifications to the study protocol must be reviewed and approved by the Ethics Committee of the Sixth Affiliated Hospital of Sun Yat-sen University. Additionally, such amendments will be updated in the Chinese Clinical Trial Registry to ensure public transparency and regulatory compliance.

### Dissemination plans {31a}

The trial results will be published in relevant journals.

## Discussion

The primary objective of this study is to evaluate the efficacy and safety of HI-IMT using the POWERbreathe KH2 device in patients undergoing tracheostomy. Although HI-IMT has shown promising results in various clinical populations, including those with chronic obstructive pulmonary disease, heart failure, and mechanically ventilated patients [16, 31, 46], there is limited evidence regarding its use in tracheostomised patients. When considering the implementation of IMT in this population, the choice of training intensity is a key consideration. In clinical practice, IMT regimens primarily comprise two strategies: high-intensity, low-repetition protocols aimed at improving muscle strength and low-intensity, long-duration protocols focused on enhancing endurance [23, 47, 48]. Since tracheostomized patients are often critically ill and possess a limited capacity for sustained inspiratory effort, high-intensity, low-repetition protocols may be more suitable.

This study is expected to provide valuable insights into the application of HI-IMT for improving diaphragmatic function, airway clearance, and overall clinical outcomes among tracheostomized patients. These results may support the development of standardized respiratory rehabilitation protocols tailored to this high-risk population. Nevertheless, several limitations should be acknowledged. First, the single-center design may restrict the generalizability of the findings to broader clinical settings. Second, while the 2-week intervention period is consistent with previous IMT trials [24, 28], it does not allow for assessment of long-term outcomes or durability of the intervention effects. Third, our study design does not include a low-intensity IMT group, which limits our ability to draw conclusions regarding the optimal training intensity for this population. Fourth, the impossibility of blinding the physiotherapists who will administer the training introduces the risk of performance bias, although this is partially mitigated through the blinding of outcome assessors and statisticians.

Future studies should aim to address these limitations by conducting multicenter trials with larger sample sizes to validate and extend the present findings. Prolonging the intervention duration and incorporating long-term follow-up would help assess the sustainability of the observed benefits. Further investigations should also directly compare different training intensities, examine dose-response relationships, optimize rest intervals, and explore personalized protocols.

## Trial status

The current protocol corresponds to version 2.0. Recruitment for the trial began in August 2025, and the trial is expected to be completed in August 2026.

## Supplementary Information

Supplementary Material 1

Supplementary Material 2

## Data Availability

The final trial dataset will be available upon reasonable request from the corresponding author.

## Abbreviations

ACBT: Active cycle of breathing technique
CONSORT: Consolidated Standards of Reporting Trials
CRF: Case report form
DMC: Data Monitoring Committee
HI-IMT: High-intensity interval inspiratory muscle training
IMT: Inspiratory muscle training
ITT: Intention-to-treat
MIP: Maximal inspiratory pressure
NMES: Neuromuscular electrical stimulation
PIF: Peak inspiratory flow
PP: Per-protocol
S5Q: Standardized Five Questions
SAS: Self-Rating Anxiety Scale
SCSS: Semi-Quantitative Cough Strength Score
TFRL: Tapered flow resistive loading
T L: Traditional threshold loading
TQOL: Tracheostomy-Specific Quality of Life
TSC: The trial steering committee
