# Supplementary material for: Effects of high-intensity interval inspiratory muscle training on diaphragm function and quality of life among tracheostomized patients: study protocol for a randomized controlled trial
Source: Trials. 2025 Dec 3;27:9. doi: 10.1186/s13063-025-09334-3 (PMC12781243; doi:10.1186/s13063-025-09334-3)
Supplement: Supplementary file 1 — Supplementary Material 1 [file 13063_2025_9334_MOESM1_ESM.pdf]

Informed Consent Form

Protocol ID: HI-MIT20240709

Informed Consent Form Version: 2.0

Protocol Title: Effects of high-intensity interval inspiratory muscle training on diaphragm function and quality of life among tracheostomized patients: Study protocol for a randomized controlled trial

Protocol Number: HI-MIT20240422

Protocol Version: 2.0

Informed Consent Form Version: 2.0

Version Date: July 9, 2024

Research Institution: Department of Rehabilitation Medicine, The Sixth Afliated Hospital, Sun Yat-Sen University

Principal Investigator (PI): ___________________________

Investigator Signature: ___________________________

Please Read the Following Information Carefully

You are being invited to participate in a clinical research study. This informed consent form provides information to help you decide whether to participate. Please read it carefully. You may ask the research investigator any questions at any time, and they will provide detailed answers. You may take time to consider your decision based on your personal circumstances.

1. Study Purpose

Background: Tracheostomy is a critical clinical intervention for critically ill patients. However, the associated complications and the specialized care required for long-term tube placement impose significant burdens on patients, their families, and society. Tracheostomies can interfere with normal diaphragmatic movement and disrupt the physiological negative-pressure breathing mechanism. Furthermore, these patients often experience severe conditions, prolonged bed rest, reduced chest wall mobility, decreased respiratory muscle strength, and are prone to atelectasis or aspiration pneumonia. Therefore, respiratory muscle training is crucial for improving patient outcomes. Currently in clinical practice, respiratory training for tracheostomy patients is mainly based on conventional methods such as abdominal (diaphragmatic) breathing, pursed-lip breathing, candle blowing exercises, paper strip resistance exercises and the use of incentive spirometers or simple respiratory training devices. However, these methods cannot accurately assess respiratory muscle strength or provide individualized training plans, meaning they cannot precisely set or maintain training loads. Additionally, like limb muscles, respiratory muscles are skeletal muscles; therefore, strength training requires the principle of progressive overload. Therefore, these non-resistance breathing exercises may not sufficiently enhance pulmonary function. Therefore, seeking additional, more targeted respiratory training methods may be beneficial for patients.

Inspiratory muscle training (IMT), which targets the diaphragm and accessory inspiratory muscles, has demonstrated efficacy in mechanically ventilated populations. However, the existing evidence is predominantly focused on intubated populations, with sparse data specific to tracheostomized patients. High-intensity interval inspiratory muscle training, characterized by cyclic phases of high-resistance loading ($\geq$50% MIP) interspersed with programmed recovery intervals, may better align with the principles of skeletal muscle adaptation.

Study Objective: The objective of this study is to evaluate the effects of high-intensity interval inspiratory muscle training(HI-IMT) on diaphragmatic function, airway clearance ability, anxiety levels, decannulation rates, quality of life, and safety outcomes in tracheostomized patients.The diaphragmatic function, airway clearance ability, anxiety levels, and quality of life will be evaluated using the Maximum Inspiratory Pressure (MIP) and Peak Inspiratory Flow (PIF), Semi-Quantitative Cough Strength Score (SCSS), Standardized Zung's Self-Rating Anxiety Scale (SAS), and Tracheostomy-specific Quality of Life (TQOL) scale. Furthermore, extubation rates and the incidence of adverse events will be meticulously documented.

2. Study Procedures

If you agree to participate, you will be assigned a unique identification number and randomly allocated to one of two groups: the HI-IMT Group or the Control Group. A medical record will be established for you. The study expects to enroll 70 participants (35 per group). Each session will last approximately 20 minutes.

Using the HI-IMT Group as an example:

Baseline Assessment: You will complete a questionnaire about your personal information. Please answer honestly.

MIP & PIF Measurement: A trained therapist will assess your diaphragmatic function using a portable pulmonary function device. During the assessment and training: Your tracheostomy tube cuff will be inflated. A Heat and Moisture Exchanger will be used as an adapter, connecting your tracheostomy tube to the breathing trainer.

Training Familiarization: Before the formal assessment, you will practice using the device 5-7 times in the "Breath Strength Test" mode, while observing the curve display.

MIP Measurement: You will perform the MIP test: exhale slowly for at least 6 seconds to approach residual volume, then inhale maximally and sustain the effort for at least 1.5 seconds. This will be repeated at least 3 times with 2-minute rests between attempts. The best value will be recorded as your MIP. PIF will be measured in a similar manner.

Other Assessments: You will also complete other functional assessment scales.

HI-IMT Intervention: The physiotherapist will prescribe an intensity of 50% of MIP for the first training set, increasing by 2 cmH2O per day, based on your tolerance. The breathing pattern involves fast, maximal effort inhalation followed by slow exhalation. Each session consists of 30 breath cycles, divided into 5 sets of 6 breaths, with 2-minute rests between sets. Training occurs once daily, 5 days per week, for 2 weeks.

Safety Monitoring: Vital signs (heart rate, blood pressure, oxygen saturation, respiratory rate) and perceived exertion (using the Modified Borg Scale) will be recorded before and after each training session.

The control group will follow the same evaluation protocol and training program but will receive a different level of resistance.

3. Study Risks and Discomfort

Current research indicates that supervised HI-IMT is generally safe and unlikely to cause adverse reactions. However, as the training involves an inspiratory load, you may experience sensations of inspiratory effort. Some patients may feel dizzy after training, but this usually resolves with rest. You can communicate any discomfort to the research staff at any time; they can make adjustments to minimize it. If you experience intolerable discomfort, you may withdraw from the study at any time. All your information will be kept confidential and used solely for this research.

4. Potential Benefits of Participation

You will receive professional assessment and training tailored to your respiratory function status, free of charge. Your participation may contribute valuable knowledge that benefits the rehabilitation of future tracheostomized patients.

5. Costs and Compensation

Research-Related Costs: You will not incur any costs for participating. All study-related tests and procedures will be provided free of charge.

Compensation: You will not receive financial compensation for participation.

6. Participant Responsibilities

- Provide truthful information about your medical history and current health status.
- Inform the research doctor about any discomfort experienced during the study.
- Inform the research doctor if you have recently participated in, or are currently participating in, any other research studies.

7. Voluntary Participation

Your participation is entirely voluntary. You may choose not to participate or withdraw from the study at any time by notifying the investigator. Your decision will not affect your current or future medical care or rights in any way. The research doctor may discontinue your participation if you require other treatment, do not follow the study plan, experience a study-related injury, or for any other reason deemed necessary.

8. Privacy and Confidentiality

If you participate, your identity and all personal information obtained during the study will be kept confidential. Your functional data will be identified only by your study number, not your name. Personally identifiable information will not be disclosed to anyone outside the research team without your permission, except as required by law or regulation. All researchers and sponsors are obligated to maintain confidentiality. Your records will be stored in locked cabinets accessible only to authorized research personnel. Representatives of government regulatory authorities or the Institutional Review Board/Ethics Committee may inspect your records to ensure the study is conducted properly. If results of this study are published, your personal identity will not be revealed.

9. Management of Research-Related Injury

If you suffer an injury directly resulting from participation in this research, you will receive necessary medical treatment. Compensation for any harm and associated medical expenses incurred due to research participation will be provided in accordance with Chinese law.

10. Other Information

This study protocol and informed consent form have been reviewed and approved by the Institutional Review Board / Independent Ethics Committee (IRB/IEC) of our institution.

If you have concerns about any aspect of this study or believe your rights as a participant have been violated, you may contact the Hospital Ethics Committee directly.

Phone: +86-20-38379764

Email: zslyllb@mail.sysu.edu.cn

If you voluntarily agree to participate, your signature on this form indicates that you understand the information provided and consent to participate. You may inquire about study-related information and progress at any time. For questions about the study, any discomfort or injury experienced during participation, or concerns about participant rights, please contact: **Fangting CHEN at +86 15927483016**

PARTICIPANT INFORMED CONSENT SIGNATURE PAGE

I have read this informed consent form.

I have had the opportunity to ask questions, and all my questions have been answered.

I understand that participation in this study is voluntary.

I understand that I may choose not to participate or withdraw from the study at any time without prejudice or loss of benefits to which I am otherwise entitled. My medical care will not be affected.

I understand that the investigator may discontinue my participation if I require other treatment, do not follow the study plan, experience a study-related injury, or for any other reason.

I will receive a signed copy of this informed consent form.

Participant's Name (Print): ________________________

Participant's Signature: ________________________ **Date:** ______

Legally Authorized Representative's Name (Print): ________________________

Legally Authorized Representative's Signature: ______________________

Relationship to Participant: ________________________ **Date:** ______

Statement of Person Obtaining Consent:

I have accurately explained the nature, purpose, procedures, potential risks, benefits, and alternatives to participation in this study to the participant/legally authorized representative. I have answered all questions. I confirm that the participant/representative voluntarily agrees to participate.

Investigator's Name (Print): __________________________

Investigator's Signature: __________________________ **Date:** ______

Ethics Approval Number: ______________
